# Disseminated Ureaplasma infection: A case report of septic polyarthritis in a patient on Rituximab therapy

**DOI:** 10.1016/j.idcr.2024.e02101

**Published:** 2024-10-18

**Authors:** Michael Axenhus, Jesper Ericson, Agata Rysinska, Annelie Petterson, Desiree Friis

**Affiliations:** a Danderyd Hospital, Department of Orthopedic Surgery, Stockholm, Sweden; b Danderyd Hospital, Department of Infectious Diseases, Stockholm, Sweden; c Department of Clinical Sciences, Danderyd Hospital, Karolinska Institutet, Stockholm, Sweden

**Keywords:** Disseminated Ureaplasma, Immunocompromised patient, Rituximab treatment, Septic arthritis

## Abstract

**Introduction:**

Immunocompromised individuals, such as those undergoing Rituximab therapy, are susceptible to severe infections by these organisms. We present a rare case of polyarticular septic arthritis caused by disseminated Ureaplasma urealyticum in a Rituximab-treated patient.

**Presentation of case:**

A 38-year-old male with a history of schizophrenia and multiple sclerosis presented with intense pain, swelling, and fever, along with limited joint mobility. Despite initial treatment with antibiotics and surgical intervention, the patient's condition deteriorated. PCR assays confirmed the presence of Ureaplasma urealyticum, prompting a change in antibiotic therapy. With focused antimicrobial treatment and supportive care, the patient exhibited gradual improvement, although reinfection occurred one month after discharge, necessitating additional surgical interventions and antibiotic therapy.

**Discussion:**

Septic arthritis due to Ureaplasma urealyticum is exceedingly rare but can occur in immunocompromised patients undergoing Rituximab therapy. Accurate pathogen identification using PCR assays is crucial for optimizing therapeutic outcomes in such cases. Treatment typically involves a combination of surgical debridement and tailored antimicrobial therapy with agents effective against Ureaplasma species. Close monitoring for disease recurrence and joint function is essential for long-term management.

**Conclusion:**

This case highlights the diagnostic challenges and therapeutic complexities of septic arthritis caused by Ureaplasma urealyticum in immunocompromised patients undergoing Rituximab treatment. Interdisciplinary collaboration and the use of PCR assays for accurate pathogen identification are crucial for successful outcomes in such cases. Clinicians should consider the unique susceptibility of immunocompromised individuals to rare pathogens and tailor antimicrobial therapy accordingly.

## Introduction

*Ureaplasmas*, which include *Ureaplasma urealyticum* and *Ureaplasma parvum*, are common pathogens in the human urogenital tract and are associated with various urogenital infections [1], [2]. Ureaplasma species are part of the normal bacterial flora in the genital microbiota and there is a high prevalence of colonization in the genital tract [3]. However, Ureaplasma can cause extragenital and disseminated infections and can go undiagnosed due to their rarity outside of the genital tracts [4], [5]. Sever infections of ureaplasma can occur in immunocompromised individuals, such as those with hypogammaglobulinemia, hematologic malignancies, or solid organ transplants [6], [7], [8].

We present a rare case of polyarticular septic arthritis caused by disseminated *Ureaplasma urealyticum* in a Rituximab-treated patient. Patients with hypogammaglobulinemia might be particularly susceptible to mucosal colonization of Ureaplasma due to its ability to persist within neutrophils in the absence of antibodies [5], [9]. This vulnerability, coupled with the absence of protective mucosal antibodies, is believed to facilitate the dissemination of these organisms within neutrophils to sites with active neutrophil recruitment, such as traumatic joints.

Ureaplasma-induced septic arthritis is rare in immunocompetent patients and these observations underscore the complexity of diagnosing and treating Ureaplasma infections, especially in the context of immunosuppressive therapies. This case report is reported in line with the SCARE criteria [10].

## Case

The patient, a 38-year-old male, was admitted to our hospital with a history of schizophrenia and multiple sclerosis (MS). Initial presentation was associated with intense pain and swelling in the left knee and complaints of a tender right shoulder, along with a high-grade fever and limited joint mobility over the past week.

The patient had a history of schizophrenia managed with antipsychotic medications and was diagnosed with relapsing-remitting MS 12 years ago. The patient had been on an ongoing MabThera (Rituximab) therapy for the last eight years to manage MS symptoms.

Upon admission to the orthopedic clinic, the patient reported four weeks of worsening pain, swelling, and redness in the left knee and tenderness in the right shoulder. The patient experienced difficulty moving the affected joints, accompanied by a fever peaking at 39.5 °C (103.1 °F). There was no history of recent trauma or insect bites. The patient was a smoker and had an irregular diet with suboptimal nutrition. The Body Mass Index was 15,70 kg/M^2^ at the time of admission.

During clinical examination, the patient appeared visibly uncomfortable. The left knee was warm, swollen, and erythematous. The knee joint exhibited limited range of motion, especially during extension, and showed tenderness upon palpation. No signs of skin breakdown, abscesses, or open wounds were observed.

Laboratory results indicated an elevated white blood cell count (16,800/μL) and an increased C-reactive protein level (CRP) of 250 mg/L. Blood cultures revealed the growth of coagulase-negative Staphylococcus bacteria, later defined as *staphylococcus hominis* and considered to be a contamination. Synovial fluid analysis from the left knee displayed an elevated white blood cell count of 154,000/μL. Due to the patient's immunocompromised status as a result of Rituximab therapy, septic arthritis was considered as the most reasonable diagnosis. The patient was promptly started on intravenous antibiotics (Cloxacillin 2 g × 3) and scheduled for surgical debridement and lavages.

**Fig. 1:**
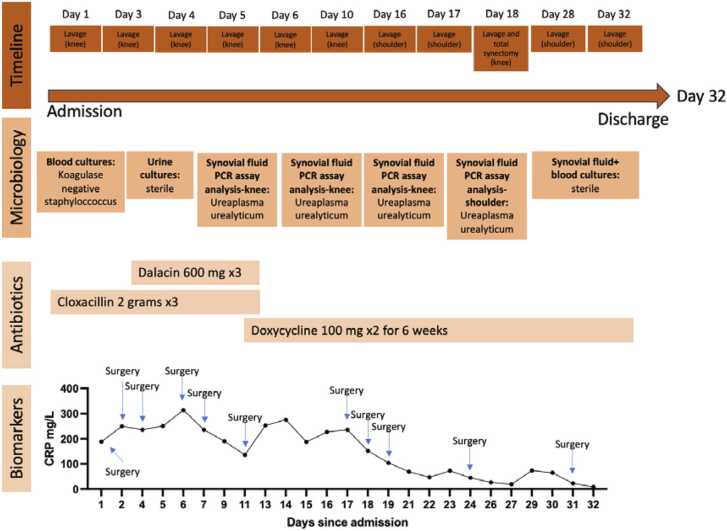
Biomarker, antibiotic treatment, microbiology, and timeline at first inpatient stay.

Despite multiple surgical debridement and lavages, the patient’s condition did not improve. CRP levels continued to rise, reaching 350 mg/L seven days after admission (Fig. 1). The patient also started complaining about swelling and pain in his left shoulder and a diagnostic arthroscopy revealed signs of infection, leading to debridement surgeries and lavages in the shoulder (Table 1).

**Table 1:** Description of total number of surgeries performed at the first inpatient stay.

Joint	Lavages	Lavage and total synovectomy
**Knee**	10	1
**Shoulder**	5	0

**Fig. 2:**
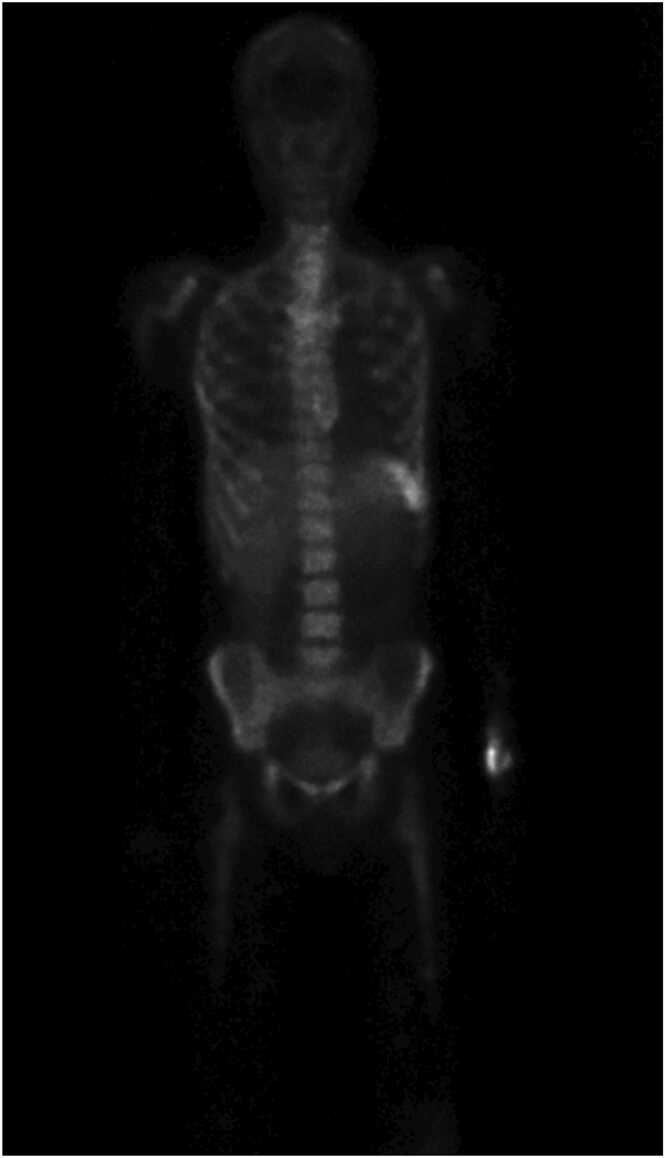
Anti-granulocyte scintigraphy showed no evidence of disseminated infection following surgeries and antibiotic treatment.

Given the patient's complicated medical history and the lack of improvement with initial treatment, a comprehensive analysis of synovial fluid was performed using S16 RNA Polymerase Chain Reaction (PCR) assays. The results confirmed the presence of *Ureaplasma urealyticum* in both knee and shoulder. In total, four different samples were PCR positive for *Ureaplasma urealyticum*. Consequently, the patient was promptly started on Doxycycline 100 mg 1 × 2, against Ureaplasma species, complemented by supportive care. An anti-granulocyte scintigraphy was conducted to explore potential additional sites of infection but yielded negative results (Fig. 2).

With focused antibiotic treatment and close monitoring, the patient exhibited gradual improvement. The fever subsided, and there was a significant reduction in joint swelling and pain. Physical therapy was initiated to restore joint mobility and function, addressing considerable extension deficit in the left knee and shoulder. Upon discharge, the patient received an extended course of Doxycycline and was scheduled for follow-up appointments to monitor joint health and overall recovery.

The patient returned for a follow-up visit approximately one month after discharge. The patient showed good mobility in both joints and had regained some muscle strength. CRP level was 9 mg/L. An electrophoresis analysis showed normal levels of IgA and IgG but undetectable levels of IgM indicating the presence of memory B-cells capable of IgG production but unable to produce new IgM. Doxycycline was therefore discontinued.

**Fig. 3:**
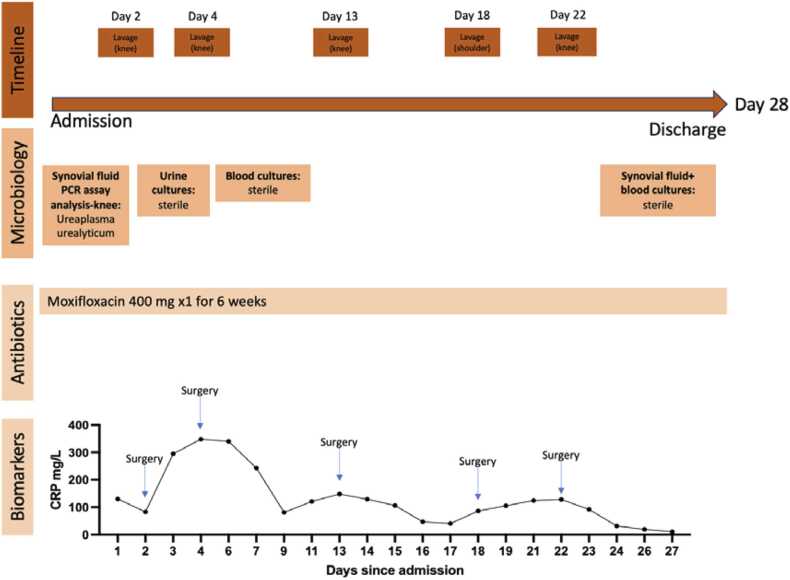
Biomarker, antibiotic treatment, microbiology, and timeline at second inpatient stay.

One month later, the patient presented with knee swelling. A new diagnostic arthroscopy in the knee confirmed reinfection of *Ureaplasma urealyticum*. The patient underwent an additional 5 debridement and lavages of the knee (Fig. 3). Antibiotic treatment was changed from Doxycycline to Moxifloxacin 400 mg 1 × 1. After a total of 6 weeks of Moxifloxacin treatment, the Moxifloxacin was discontinued following an outpatient clinical and biochemical examination. The patient continued to improve in joint mobility and did not experience any additional symptoms at a follow up 6 months later.

## Discussion

Septic artrithis due to *Ureaplasma urealyticum* is exteedingly rare and present several clinical considerations. Immunocompromised patients are at risk for infection and may be prone to infections by rare pathogens. Good clinical outcomes can be achieved with adequate antimicrobial treatment and proper surgical intervention. Physicians should consider the benefit of early multidisciplinary approach in treating patient with septic artrithis due to *Ureaplasma urealyticum.*

Rituximab, which can cause B-cell depletion and secondary hypogammaglobulinemia, is a common drug used to treat MS. Patients undergoing Rituximab treatments can therefore be considered more vulnerable to infections [11], [12]. Although rare, joint infection by Ureaplasma species in patient with ongoing Rituximab treatment represent clinical challenges and clinicals should consider the potential of unusual pathogens in immunocompromised individuals.

Our case echoes the diagnostic hurdles elucidated in prior studies. Ureaplasma are facultative anaerobic bacteria and are therefore notoriously difficult to detect using standard culture media. This fact was noted in our case as initial routine blood and synovial fluid cultures did not display Ureaplasma species.

Septic arthritis due to Ureaplasma is exceedingly rare and few cases have been described in the literature with a focus on patients with immunodeficiencies, prolonged B-cell depletion and secondary hypogammaglobulinemia [13]. A case of septic arthritis caused by Ureaplasma have been described in juvenile idiopathic arthritis [14]. Septic arthritis caused by Ureaplasma species can cause sepsis and represent a diagnostic challenge for physicians [15]. Furthermore, septic arthritis caused by *Ureaplasma urealyticum* has mainly been described in females rather than males [8], [16], [17]. Lastly, a case of *Ureaplasma urealyticum* has been reported in hip arthroplasty as the causative agent of post operative infection requiring revision [18].

The use of S16 RNA PCR assays played a pivotal role in identifying *Ureaplasma urealyticum* as the causative agent. PCR is not always performed during treatment for septic arthritis, with biochemical and cultures often being enough to identify a pathogen. Although, PCR assays should be considered when dealing with immunocompromised patients due to its accuracy in identifying unusual pathogens. Previous reports have shown that Ureaplasma species can be accurately detected utilizing PCR with a high degree of sensitivity, making this a useful method of choice for microbial identification in immunocompromised individuals [9], [19].

In this case, the patient exhibited a favorable response to appropriate antibiotic and surgical treatment, signifying the significance of accurate pathogen identification in optimizing therapeutic outcomes. Surgical debridement and lavage by itself is not enough to ensure proper management of septic arthritis caused by *Ureaplasma urealyticum* and clinicians should take care in the selection of proper antimicrobial drugs.

In terms of antimicrobial treatments, clinicians should consider macrolides and quinolones, which should prove effective against all Ureaplasma species. It should be noted that ureaplasma lack a peptidoglycan cell wall, reducing the effectiveness of folic acid inhibits and β-lactams, making these unsuitable as treatment options [20]. Septic arthritis is an acute condition, requiring rapid intervention to save joint and limb. In the event of unknown antimicrobial susceptibility, a common clinic problem, two antibiotics might be more beneficial compared to monotherapy [9], [20].

Patients such as ours, with underlying conditions necessitating immunosuppressive treatments, require meticulous long-term monitoring. Post-treatment, close surveillance for disease recurrence, joint function, and overall well-being is crucial. We treated the patient successfully with Doxycycline initially and eventually a 6 weeks course of Moxifloxacin. The treatment time should be individualized and determined by clinical response and severity of infection.

## Conclusion

In conclusion, our case highlights the diagnostic challenges and therapeutic complexities of septic arthritis caused by *Ureaplasma urealyticum* in immunocompromised patients undergoing Rituximab treatment. Through correct diagnostic techniques, interdisciplinary collaboration, and tailored antimicrobial therapy, successful outcomes can be achieved. Clinicals should consider utilizing PCR assays in the event of join infections in immunocompromised individuals.

## CRediT authorship contribution statement

**Annelie Petterson:** Data curation, Formal analysis, Investigation, Writing – review & editing. **Desiree Friis:** Data curation, Investigation, Validation, Writing – review & editing. **Michael Axenhus:** Conceptualization, Data curation, Formal analysis, Investigation, Methodology, Resources, Software, Writing – original draft. **Jesper Ericson:** Conceptualization, Data curation, Formal analysis, Investigation, Writing – review & editing. **Agata Rysinska:** Data curation, Formal analysis, Investigation, Software, Writing – review & editing.

## Ethical approval

None.

## Declaration of Competing Interest

The authors declare no competing interests.
